# A training programme for novice extracorporeal resuscitation providers

**DOI:** 10.1016/j.resplu.2024.100720

**Published:** 2024-07-17

**Authors:** Natalie Kruit, Aidan Burrell, Casey Edwards, Mark Dennis

**Affiliations:** a FANZCA: NSW Ambulance. Faculty of Medicine and Health, University of Sydney, Department of Perioperative Medicine, Westmead Hospital, Australia; b Australian and New Zealand Intensive Care Research Centre, Monash University, Melbourne, Vic., Australia; c The Alfred Hospital, Melbourne, Victoria, Australia; d Australian Blood Management, University of Western, Sydney, Australia; e Department of Cardiology Royal Prince Alfred Hospital, Faculty of Medicine and Health, University of Sydney, Australia

**Keywords:** Venoarterial extracorporeal membrane oxygenation (VA ECMO), Extracorporeal life support (ECLS), Pre-hospital, extracorporeal cardiopulmonary resuscitation (E-CPR), Advanced cardiopulmonary resuscitation, Training, Education

## Abstract

**Introduction:**

The use of extracorporeal cardiopulmonary resuscitation (ECPR) for refractory cardiac arrest is increasing globally. However, providing equity of access to all patients is challenging, and to date, access has been limited to inner city areas surrounding major hospitals. To increase the availability of ECPR in our jurisdiction, we sought to train pre-hospital physicians with no experience in extracorporeal membrane oxygenation cardiopulmonary resuscitation (ECPR). To enable this, we sort to develop and teach a syllabus that would provide novice ECPR providers the skill to perform ECPR safely and effectively in the pre-hospital environment.

**Methods:**

This training programme consisted of 11 pre-hospital physicians and six critical care paramedics. All participants had no prior hospital experience instituting or managing ECPR patients. The training programme was multimodal utilising a porcine model of heart failure to teach time pressured dynamic physiological troubleshooting, cadaver labs to teach cannulation, didactic teaching and simulation. Key knowledge and skill domains were identified. Each learning framework was built upon with a final focus on integrating all skill domains required to successfully initiate ECPR.

**Results:**

The training program was completed from February 2022 to August 2023. Knowledge progression was assessed at key stages via written and practical examination. Each participant demonstrated clear knowledge and skill progression at the key stages of the training programme. At the end of the training programme, participants met the pre-defined standards to progress to ECPR provision in the pre-hospital environment.

**Conclusion:**

We present a training program for novice ECPR providers performing ECPR in the pre-hospital setting. The outcomes of this training program can provide a training framework for both novices, low volume ECMO centres and pre-hospital clinicians.

## Background

The use of extracorporeal cardiopulmonary resuscitation (ECPR) for refractory cardiac arrest is increasing.^1^, ^2^ The delivery of ECPR is complex, resource intensive, technically challenging and time pressured. Owing to these challenges, provision of ECPR has historically been completed, where possible, on arrival to an ECMO capable hospital.

Outcomes of ECPR are significantly influenced by “low flow” time, the time from commencement of cardiopulmonary resuscitation (CPR) to establishment of extracorporeal flow. It is recommended that ECPR is implemented within 60 min of cardiac arrest^3^, ^4^, ^5^ and the best survival rates are within 30 min.^6^, ^7^ Owing to obligate ambulance response times, initial patient resuscitation, extrication and transportation back to hospital, it is challenging to minimise low flow time. Even in well-established cardiac arrest systems and recent clinical trials, establishing ECPR in under 60 min is challenging.^8^, ^9^, ^10^ Delivery of ECPR in the community – pre-hospital ECPR − may reduce low flow time and increase the geographical reach of an ECPR service above hospital based ECPR strategies^8^, ^11^ thereby improving equity of access to ECPR.

Pre-hospital ECPR services/trials are increasingly being offered; including in Paris,^12^ London,^13^ Melbourne,^14^ Albequerque,^15^ Regensburg^16^ and the Netherlands (NCT04620070). The composition and training of the pre-hospital ECPR team vary and are not well described. A pre-hospital team, compromising of a specialist doctor and critical care paramedic is available 24/7 in our jurisdiction thereby allowing for a rapid and consistent response to cardiac arrest. In addition to this, the Ambulance service is staffed with intensive care paramedics who are usually the highest clinical level attending a cardiac arrest, it is within their skill set to provide advanced interventions such as intubation. The intent of this model is to task the Paramedic team with provision of advanced cardiac life support whilst the medical team focused on ECPR. Training pre-hospital specialists in ECPR would potentially provide a model of ECPR service delivery that would reduce geographical challenges and transport times, low flow time, and potentially improve patient outcomes.

In order to facilitate our pre-hospital ECPR feasibility study, we sought to train a group of pre-hospital physicians and critical care paramedics to perform ECPR for the first time in the pre-hospital environment. Management principles of V-A ECMO and ECPR were limited to the first four hours post initiation to enable safe transfer to a high volume ECMO centre with expertise.

## Methods

### Participants

Training participants consisted of 9 emergency physicians, 2 anaesthetists and 6 critical care paramedics who were employed by the state aeromedical retrieval service. Physicians pre-hospital experience ranged from 10-25 years, paramedic experience was greater than 20 years. All participants had limited or no prior experience in ECMO or ECPR.

## Training program structure

The key components of and the core competencies required for successful ECPR cannulation and management of the patient in the first four hours following ECMO initiation were identified^17^ and supplemented via an international Delphi process.^18^ These domains included theoretical knowledge, vascular access and management of extracorporeal support (Table 1). The structure of the training was divided into 2 distinct phases; stage 1: theoretical and practical skill acquisition in a simulated environment (Table 1); stage 2: in vivo learning/experience of pre-hospital ECPR initiation with mentorship from an ECMO specialist (Table 2). The training programme was supported by a faculty of experienced ECMO providers which included cardiothoracic surgeons, a vascular surgeon, clinical perfusionists, cardiac anaesthetists and intensivists who defined the required standards to be met for independent practice. Candidates were required to pass all assessments and be independently signed off by one of the expert faculty prior to entering stage 2.

**Table 1:** Stage 1 training programme and competency assessment. Training and learning Framework.

	**Learning Outcomes**	**Training**	**Proficiency Requirement**
**Theory**	Foundational knowledge pertaining to: Components of circuit and membrane oxygenation Console, circuit and pump emergencies Physiology of VA ECMO Principles of managing VA ECMO Principle of cannulation including femoral anatomy Patient selection in ECPR Principles of managing an ECPR patient in the first 4 hours	10hr online learning module 6 days face-to-face didactic teaching	Pre and post written exam after each learning module ASSESSMENT: Score > 80% on final written exam
**Ultrasound**	a) Identifying femoral sono-anatomy in the arrested patient b) Needle tip visualisation c) Ability to confirm wire in the vessel with IVC and aorta imaging d) Basic cardiac TTE focusing on haemodynamic assessment and AV opening	a) Cadaver lab b) Part task trainers c) Regular imaging IVC/Aorta d) Regular TTE practice	Clips of 5 IVC/aorta images Formative and summative assessment of ultrasound guided vascular access
**Cannulation**	a) Perform regular femoral arterial vascular access (sheath insertion) during course of training b) Identify common femoral artery and vein in arrested patient utilising surface and sono-anatomy c) Perform the steps of ECMO cannulation d) Progression through varying levels of technical difficulty and demonstrate an understanding of trouble shooting ECMO cannulation issues, e.g. kinked wire	a) Vascular access lists e.g. coronary angiography, interventional radiology b) Cadaver labc) Repetitive skill exposure with expert feedback (part task trainer) d) Modification of part task trainer to increase technical difficulty	a) Log-book attendance c) Graded progression of micro-skills as competency achieved: • Identify anatomical landmarks for CFA/V vessel puncture • U/S guided femoral vascular access, needle tip visualised, shallow angle of approach (45 degrees) • Needle control whilst wire being fed • Visualisation of wire in vessel • Serial vessel dilation: i) Control of neurovascular bundle, minimise bleeding ii) Wire control ensuring awareness of wire depth, benching wire as dilator inserted iii) Confirm wire moves freely prior to advancing or withdrawing dilator **ASSESSMENT:** Score 100% before progressing to Stage 2
**Extracorporeal support**	a) Understand the principles and key components of the circuit and a centrifugal pump b) Initialisation of the pump c) Identify common issues that can occur with extracorporeal support and the management strategy d) Establish extracorporeal support in a live circulation	a) Online learning and didactic teaching b) Simulation, repeated exposure c) Online learning, didactic teaching, simulation d) Live animal circulation model	a) Pre and post written exam b) Rapid pump initialisation without using a checklist d) Independently initiates ECMO: • Achieves desired flow through manipulation of clamps, RPMs and gas flow • Effective communication throughout initiation • Identifies limitation in flow and manages appropriately
**Workflow**	Integration of all components of the ECPR process Anticipating and planning workflow for efficiency Optimisation of equipment and environment for efficient cannulation	Simulation, repeated exposure in complex environments	Successful initiation in < 20mins on mannequin model **ASSESSMENT: Score > 80%**

**Table 2:** Stage 2 training programme and competency assessment. Training and learning Framework.

	**Learning Outcomes**	**Training**	**Proficiency requirement**
**Ultrasound**	Recognition of correct sono-anatomy in the arrested patient Ability to confirm sheath wire is in vessel in the arrested patient Ability to visualise the IVC in the arrested patient and detect the presence of a single venous wire Cardiac ultrasound with basic haemodynamic assessment	In vivo learning/experience with mentorship from an expert ECMO cannulator/clinician	
**Cannulation**	a) Vessel access in an arrested patient in the pre-hospital environment b) Ability to safely insert arterial and venous ECMO cannulas under time pressure in the arrested patient	In vivo learning/experience with mentorship from an expert ECMO cannulator/clinician	a) Arterial sheath insertion in the pre-hospital setting in an arrested patient within 5 mins b) Successful VA ECMO cannulation in the arrested patient in the pre-hospital environment achieving flow in under 60 minutes from time of collapse
**Extracorporeal Support**	a) Cardiohelp initialisation in a time pressured environment with multiple distractors b) Circuit management: safe and effective manipulation of circuit flow, sweep gas flow, volume c) Identifying common issues associated with the circuit and trouble-shooting accordingly	In vivo learning/experience with mentorship from an expert ECMO cannulator/clinician	a) Cardiohelp initiation executed with accuracy and efficiency ensuring: • Gas delivery line connected and turned on • Heparin administered • Lines clamped in correct position b) Independent initiation of ECMO, successfully achieving the maximum flow possible for a given patient • Is not reliant upon prompts or aids • Communicates effectively throughout ECMO initiation • Identifies key concerns immediately • Identifies when stable, maximal flow is achieved c) Demonstration of understanding of requirements to achieve stable flow

The structure of the programme followed a mastery based learning framework, employing the following key components: (a) pre-existing (baseline) assessment for all trainees; (b) identified core competencies taught in a graded fashion; (c) use of deliberate practice (d) minimum standard set for achieving competency with continued practice required until competency reached.^19^ Each learning framework was built upon with a final focus on integrating all knowledge and skill domains required to successfully initiate ECPR.

### Stage 1

#### Theoretical knowledge

The principles of extracorporeal support, including the components of the circuit and membrane oxygenator, physiology of veno-arterial (VA) ECMO, management of patients supported with VA ECMO, evidence for ECPR indications, femoral anatomy and sono-anatomy and management specific to ECPR patients were taught via a 10 h online learning module and face to face lectures (Table 1). Participants undertook an exam pre and post these learning modules to track progress and modify the education plan accordingly.

#### Vascular access and training for cannulation

**Fig. 1:**
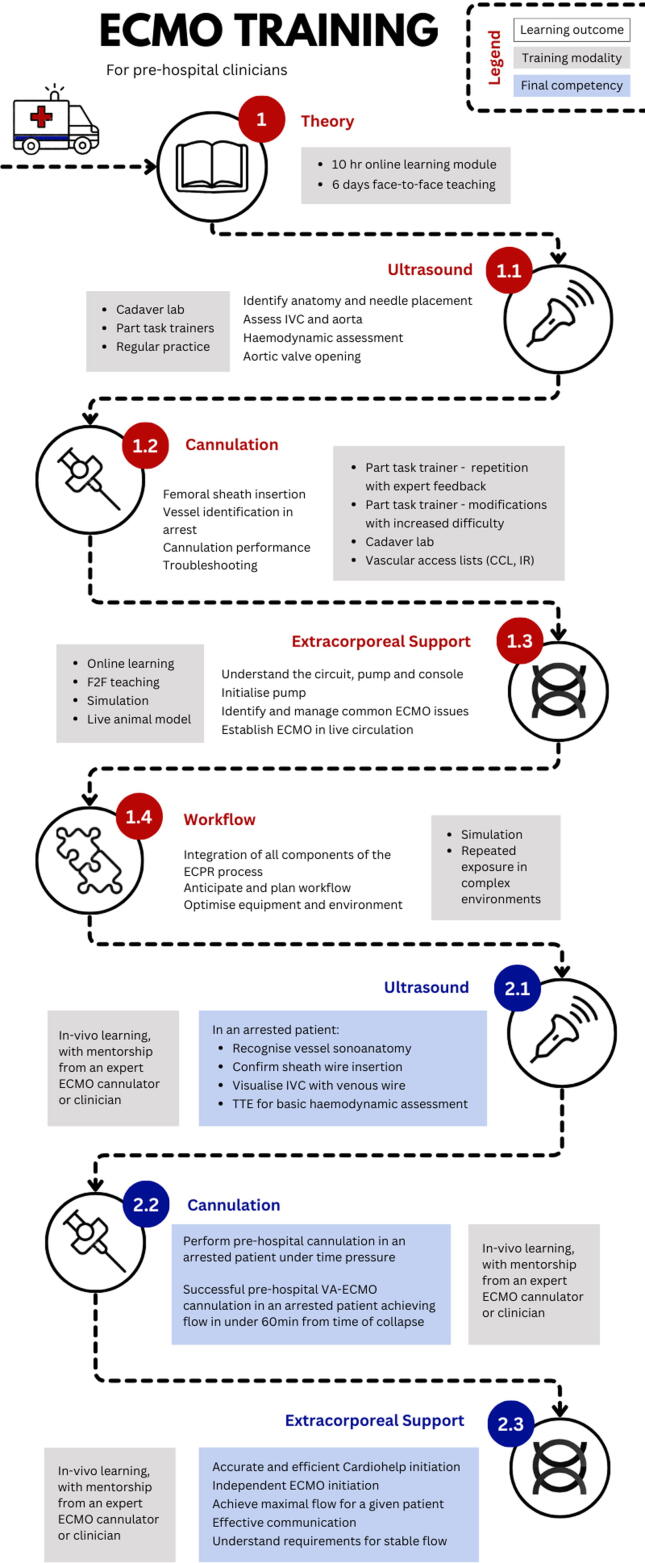
Training program and proficiency requirements.

Cannulation was taught using multiple simulation modalities including part task trainers and cadaver labs (Table 1^,^ Fig. 1). Foundational cannulation skills were taught utilising a part task trainer. The cannulation process was broken down into “micro-skill” segments (Table 1) with graded progression to the next skill level. Once the process had been successfully mastered, the part task trainers were modified to increase technical difficulty allowing the trainees the opportunity to progress through varying levels of difficulty.

The cadaver lab provided foundational knowledge of anatomy and sono-anatomy and the subsequent application during vascular access in an arrested patient. Femoral cut down was performed under the tuition of a vascular surgeon to reinforce anatomical landmarks and anatomical variations (not as a cannulation technique which is to be Seldinger based). The cadaver lab provided participants with the experience of cannulating human tissue and to demonstrate the issues that can occur with normal human anatomical variation.

Cannulation training occurred monthly over a 12-month period and in keeping with the evidence from resuscitation education and training, the skill of cannulation was taught utilising deliberate practice repetitive skill exposure combined with expert feedback.^20^, ^21^, ^22^, ^23^ Participants skill progression and learning requirements were assessed on a regular basis with formative assessment shaping the progression and content of the cannulation training programme (Table 1^,^Fig. 1). The final summative assessment required participants to successfully perform a cannulation on a part task trainer, demonstrating the micro skills identified for competent and safe practice (Table 1, appendix 1). Assessment of proficiency was completed by existing ECMO experts (BP, NK).

#### Managing an extracorporeal circuit

Teaching of the management of the extracorporeal circuit was completed using a multi-modal approach; including didactic lectures, simulations and live circulation animal models.

The live circulation animal model ECMO laboratory (LCAMEL) was designed to provide trainees with experience establishing extracorporeal support in a live circulation in a controlled environment prior to initiating ECMO for the first time in the pre-hospital setting. The porcine model was used to consolidate theoretical learning and provide context for dynamic management concepts, assisting in the application of knowledge throughout a variety of haemodynamic conditions. In brief, ventricular dysfunction was pharmacologically induced prior to ECMO initiation. After echocardiographic confirmation of severe ventricular impairment, ECMO was initiated and the haemodynamic profile was manipulated to affect extracorporeal flow thereby exposing participants to predefined haemodynamic abnormalities and pump emergencies seen in ECMO support (Appendix 2). In real time, participants were required to identify the circulatory problem and rectify the issue promptly. Participants were exposed to 4 sessions in the lab. On completion, participants were expected to be able to independently initialise the pump, safely establish target ECMO flow and if target ECMO flow could not be reached, identify the cause of flow limitation and treat appropriately. Each session increased in difficulty with decreasing levels of supervision culminating in a requirement for no assistance by the final assessment (Appendix 3).

#### Simulation training

Once foundational learning outcomes and competencies had been achieved, wholistic simulation training was used to integrate all knowledge and skill components into a seamless process necessary for ECPR initiation (Table 1). Eight simulations were completed by each participant (Fig. 1). In addition to regular wholistic simulation, core skills essential to ECPR initiation e.g. wet connection, console initiation, running on to ECMO, were identified and rehearsed to the point of automation to facilitate speed and reduce cognitive load.

### Stage 2

In order to progress to stage 2 of the training programme, clinicians were required to demonstrate proficiency in all domains outlined in Table 1. Stage 2 comprised of in vivo cannulation experience with an experienced ECMO provider. Clinicians were required to rapidly achieve femoral sheath access, successfully prepare equipment and assist in an ECPR cannulation and perform an ECPR cannulation demonstrating all skills previously assessed in the simulation environment (Table 2). Once stage 2 was complete, clinicians were signed off for independent practice (Appendix 4).

#### Currency and maintenance of skill

Upon completion of the didactic teaching module, concepts learnt were reinforced with monthly cased based discussions highlighting common ECMO related issues and discussion of management strategy. All clinicians were subject to monthly currency assessing circuit and console skills with an additional requirement for the doctors to perform vascular access on the part task trainer once a shift.

#### Assessment

**Fig. 2:**
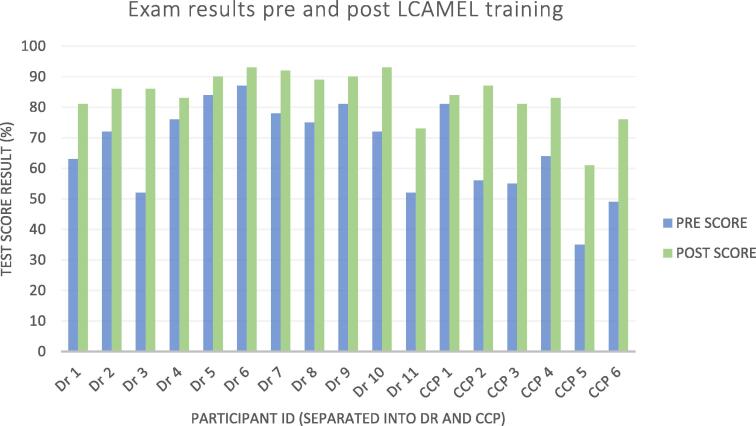
Exam results pre and post LCAMEL training.

The training program and assessment was competency based (Fig. 1). Assessment was multimodal using written, verbal and simulation based evaluations (Fig. 1, Table 1). In keeping with a mastery based framework, a baseline assessment was undertaken prior to course commencement and participants were assessed via the same written examination to track knowledge acquisition and identify learning deficiencies. Participants were also examined with a written exam before and after the LCAMEL training (Fig. 2).

At the conclusion of the training programme, participants were required to meet the standards set by the multidisciplinary faculty supporting the training programme (Appendix 4) to progress to ECPR provision in the pre-hospital environment.

## Results

On completion of the didactic course, all trainees had demonstrated significant improvement in their written exam results. Similarly, on completion of the LCAMEL live training porcine model all candidates demonstrated improved examination results (Fig. 2). Once the extracorporeal circuit training was complete, all clinicians were able to successfully demonstrate the minimum standard outlined to initialise, initiate and troubleshoot common haemodynamic issues associated with extracorporeal support. All participants met the requirements to progress to supervised ECPR provision in the pre-hospital environment.

## Discussion

We present a dedicated training program for novice ECPR providers performing ECPR in the pre-hospital setting, to our knowledge, the first of its kind published.

In an effort to expand equity of access and reduce low flow times, ECPR is now increasingly being implemented in lower volume hospitals and the pre-hospital environment.^24^, ^13^, ^25^, ^26^ Our training model enables trainees inexperienced in ECMO and ECPR provision, to meet the end goal of successfully performing ECPR cannulation in a pre-hospital environment, one of the most challenging ECPR service delivery models. Whilst it is recognised that the volume of VA ECMO experience builds expertise and is an important component of ECPR training, there is currently no evidence on the volume and duration of exposure required to competently perform ECPR cannulation.^18^

The outcomes of this training program are translatable outside of the pre-hospital environment and can provide a training framework for both novices, low volume ECMO centres and pre-hospital clinicians. Moreover, given that ECPR is a low frequency, high risk procedure, the ability to maintain volume across a number of practitioners in a scalable and sustainable service, whilst reducing low flow time and increased patient access is very challenging. Training programs such as ours offer an alternative training model providing both a syllabus and required proficiency to a achieve safe and effective ECPR provision in a low volume setting.

Whilst a number of training programs exist for ECMO support these are simulation and cannulation based without in-vivo physiology. Our porcine model used to test critical aspects of ECMO physiology, troubleshooting and time pressured decision making is unique and provides the closest dynamic learning environment next to real patient experience.

The cannulation training was spaced out over a 12-month period and was structured to ensure repeated intervals of deliberate practice to aid in skill acquisition, skill retention, improve cognitive performance, situational awareness and integration of information when performing ECPR for the first time. This educational technique has been shown to improve skill acquisition and retention^27^, ^28^, ^29^ and evidence from an ILCOR CoSTR supports the spaced structure of this program, demonstrating improvement in skill retention.^30^, ^31^

The end goal of the training program was to develop confidence in the skills acquired, training procedural and technical skills to the point of automation to facilitate speed and reduce cognitive load. This was achieved by breaking down the components of the ECPR process, training to the point of automation and then using simulation to integrate the skills and knowledge learnt ensuring the trainee could maintain situational awareness during the prehospital ECPR initiation process.^32^ There is a significant body of evidence that simulation training in ECMO improves performance and patient management.^33^, ^34^, ^35^ The success of an ECPR program depends on many factors, one of those factors is the process used for initiation. Thoroughly rehearsed, well thought out processes facilitate marginal gains in timing and ultimately lead to successful outcomes.^34^, ^36^

## Limitations

This training program occurred over a one-year period. This was due to roster restrictions. The 20 training sessions could be delivered in a shorter time frame, however providing this training in a short time frame would help prevent knowledge and skill decay potentially shortening the time required for training overall. Our current pre-hospital ECPR trial is still ongoing.

## Conclusion

We describe a training program for novice ECPR providers performing ECPR for the first time in the pre-hospital environment. Our program may provide other pre-hospital and lower volume hospital based ECPR services with a program to develop and maintain skills and knowledge of a low volume high acuity intervention.

## CRediT authorship contribution statement

**Natalie Kruit:** Writing – original draft, Project administration, Methodology, Formal analysis, Conceptualization. **Aidan Burrell:** Writing – review & editing, Supervision. **Casey Edwards:** Writing – review & editing, Conceptualization. **Mark Dennis:** Writing – review & editing, Supervision.

## Declaration of competing interest

The authors declare that they have no known competing financial interests or personal relationships that could have appeared to influence the work reported in this paper.
