# Supplementary material for: A training programme for novice extracorporeal resuscitation providers
Source: Resusc Plus. 2024 Jul 17;19:100720. doi: 10.1016/j.resplu.2024.100720 (PMC11301333; doi:10.1016/j.resplu.2024.100720)
Supplement: Supplementary Data 1 [file mmc1.docx]

|  |
| --- |

**APPENDIX 1: CANNULATION ASSESSMENT**

| **Critical Actions Checklist – ECMO 1** |
| --- |

|  |  |
| --- | --- |
| ***PLACE FEMORAL SHEATHS*** |  |
| PPE/Sterile procedures |  |
| Drape |  |
| Identify anatomical landmarks for CFA/groin puncture site |  |
| US to assess vascular anatomy: locate CFA |  |
| Stick/Wire right femoral artery:   - Shallow needle angle for puncture - Needle held in position at all times whilst wire being fed |  |
| Verify wire in vessel on ultrasound |  |
| Place 7F catheter into femoral artery |  |
| US to assess vascular anatomy: correctly locate femoral vein |  |
| Stick/Wire right femoral vein:   - Shallow needle angle for puncture - Needle held in position at all times whilst wire being fed |  |
| Verify wire in vessel on ultrasound |  |
| Place 6F catheter into femoral vein |  |
| Ultrasound IVC (subcostal) as amplatz wire being fed through arterial sheath |  |
| Recognise absence of wire in IVC as confirmation wire is arterial |  |
| Ultrasound IVC (subcostal) as amplatz wire being fed through venous sheath |  |
| Recognise wire in IVC |  |
| Situational awareness of team time out and decision to proceed with ECMO |  |
| ***CANNULATE FEMORAL ARTERY*** |  |
| Obtain dilators from CCP and wet with n/saline |  |
| Remove 7F sheath |  |
| Serial vessel dilation   - Control of neurovascular bundle above wire insertion, minimize bleeding from insertion site - Twist dilator on the way in, maintaining twist on the way out - Smooth, controlled “gliding” technique - Controlling wire as dilator comes out with clear communication as to who has hold of wire - Selection of the correct size dilators |  |
| Place arterial ECPR cannula   - Ensure obturator is clicked into cannula - Ensure cap on cannula is not lose - Ensure clamps ready/available prior to cannula insertion - Ensure cannula inserted all the way into the vessel |  |
| Remove obturator/wire, clamp |  |
| ***CANNULATE FEMORAL VEIN*** |  |
| Remove 7F sheath |  |
| serial vessel dilation with above technique |  |
| - Insert venous cannula - Remove obturator back out of cannula to identified mark when cannula inserted 30cm - Insert venous cannula 35-40cm |  |
| Remove obturator/wire, clamp |  |
| ***FLUSH/DE-AIR CANNULAE*** |  |
| Remove Red cap on arterial cannula, De-air/flush, clamp |  |
| Remove blue cap on venous cannula De-air/flush, clamp |  |
| ***GO ON PUMP*** |  |
| Call for circuit, untwist lines |  |
| Correctly clamp and divide lines at scissor mark on circuit |  |
| Identify arterial limb of ECPR circuit |  |
| Hold up limb/cannula for de-airing |  |
| Connect half-way under flush, inspect, secure |  |
| Unclamp arterial limb, leave cannula clamped |  |
| Identify venous limb of ECPR circuit |  |
| Hold up limb/cannula for de-airing |  |
| Connect half-way under flush, inspect, secure |  |
| Unclamp venous limb, leave cannula clamped |  |
| Announce sterile clamps removed, ready to commence ecmo flow |  |
| Situational awareness: dark red blood drained, bright red returned |  |

**Critical Actions Checklist – ECMO 2**

|  | SIM 1 | SIM 2 | SIM 3 |
| --- | --- | --- | --- |
| **SET UP** |  |  |  |
| Set up ultrasound |  |  |  |
| Pass drape |  |  |  |
| Hand off ultrasound cover then ultrasound probe |  |  |  |
| Open sheaths, micropuncture needle, gauze and amplatz wires |  |  |  |
| PPE/Sterile procedures |  |  |  |
| ***PLACE FEMORAL SHEATHS*** |  |  |  |
| Insert arterial and venous sheath wire as needle being held ECMO 1 |  |  |  |
| Feed amplatz wire into arterial sheath |  |  |  |
| Recognise absence of wire in IVC as confirmation wire is arterial |  |  |  |
| Feed amplatz wire into venous sheath |  |  |  |
| Recognise absence of wire in IVC as confirmation wire is venous |  |  |  |
| Situational awareness of team time out and decision to proceed with ECMO |  |  |  |
| ***CANNULATE FEMORAL ARTERY*** |  |  |  |
| Obtain dilators from CCP and wet with n/saline |  |  |  |
| Set up sterile field in ordered fashion |  |  |  |
| Pass dilators in size order placing back in tray in correct size order |  |  |  |
| Control amplatz wire ensuring end of wire stays at patient landmark (toes) and recognizing if wire migrating  Ensure racking of wire at all times with steady wire control |  |  |  |
| Clear communication as to who has hold of wire |  |  |  |
| ***GO ON PUMP*** |  |  |  |
| Call for circuit, untwist lines |  |  |  |
| Correctly clamp and divide lines at scissor mark on circuit |  |  |  |
| Identify arterial limb of ECPR circuit |  |  |  |
| Hold up limb/cannula for de-airing |  |  |  |
| Connect half-way under flush, inspect, secure |  |  |  |
| Unclamp arterial limb, leave cannula clamped |  |  |  |
| Identify venous limb of ECPR circuit |  |  |  |
| Hold up limb/cannula for de-airing |  |  |  |
| Connect half-way under flush, inspect, secure |  |  |  |
| Unclamp venous limb, leave cannula clamped |  |  |  |
| Announce sterile clamps removed, ready to commence ECMO flow |  |  |  |
| Situational awareness: dark red blood drained, bright red returned |  |  |  |

***KEY SAFTEY VIOLATIONS***

|  | SIM 1 | SIM 2 | SIM 3 |
| --- | --- | --- | --- |
| Incorrect vessel identification |  |  |  |
| Failure to confirm wires in appropriate vessels |  |  |  |
| Failure to control guidewire eg comes out, not racking the wire |  |  |  |
| Insert venous cannula all the way into vessel without obturator coming out |  |  |  |
| Cannula-vessel mismatch |  |  |  |
| Cannula-circuit tubing mismatch (e.g. Red-to-Blue) |  |  |  |
| Inadequate wet connection eg air embolus |  |  |  |
