# Supplementary material for: A training programme for novice extracorporeal resuscitation providers
Source: Resusc Plus. 2024 Jul 17;19:100720. doi: 10.1016/j.resplu.2024.100720 (PMC11301333; doi:10.1016/j.resplu.2024.100720)
Supplement: Supplementary Data 2 [file mmc2.docx]

| **Planned Manipulation** | **Means of Induction** | **Expected Management** |
| --- | --- | --- |
| **Insufficient preload**:  access insufficiency, visible chattering of access line, pVen <150mmHg, circuit flow reduced and unstable, decreased CVP and MAP in pig (relative to amount of volume removed) | Bolus dose(s) of vasodilator (GTN, propofol and isoflurane  Removal of 500-1000mL blood from the emergency prime line into blood bag as needed | Participant is expected to:  Identify access insufficiency.  Reduce circuit flows to achieve short term stability  Assess MAP if available  Administer fluid bolus  Assesses impact of intervention repeating if appropriate  Check drainage cannula position  Participant increases ECMO flow to target as soon as physiologically possible |
| **Elevated afterload:**  Increased return pressure >380mmHg  decreased circuit flow, increased MAP in pig >150mmHg | Bolus of vasoconstrictor (noradrenaline) | Participant is expected to:  Identify high afterload with impacted ECMO flows  Assess patient MAP if available  Assess return line for kinks and obstructions Requests a vasodilator and/or sedation and analgesia.  Participant persists treating afterload until ECMO flow target is achieved |
| **Hypoxia secondary to gas failure:**  Desaturated appearance of blood in access and return lines, SVO_2_ <45% | Discreet disconnection of gas delivery tubing from oxygen inlet of oxygenator  Ventilation to animal suspended | Participant is expected to:  Identify dramatic decrease in SVO_2_ and/or visible desaturation of blood in access and return lines.  Trace gas delivery system from source to oxygenator, looking for kinks or disconnections.  Observe a rise in SVO_2_ after reconnecting gas and cites that they would swap oxygen source if cause of gas failure was not obvious |
| **Air entrainment into circuit:** Visible air in the top of the oxygenator | Discreet injection of 50mL air via syringe into circuit pigtail | Participant is expected to:  Identify air, announce emergency clearly to team and clamps the arterial line  Request conventional arrest management resumes.  Divide management into “patient management” and “pump management”  Assess whether air can be removed from circuit with a syringe or whether ECMO is to be abandoned.  If air is removed, participant safely reinitiates ECMO |
| **Loss of pulsatility and LV distension:**  Decreased pulse pressure  LV distension on ECHO, minimal change to circuit pressures and flows | Heart failure induced with amiodarone and lignocaine  TOE: EF < 10%  Large volume of crystalloid administered to pig (5-7L) | Participant is expected to:  Complete ECHO and observe loss of pulsatility  Consider tamponade, LV distension, arrhythmia, valvular regurgitation and fluid overload.  Consider whether ECMO flows are excessively high and adjusts accordingly.  Assess MAP for high afterload  If poor contractility suspected, participant requests inotrope (adrenaline).  Participant continues to optimize patient without significant reduction in ECMO flow |
| **High circuit return pressures secondary to line obstruction:**  Increased return pressure >380mmHg, decreased circuit flow, no change to MAP in pig | Partial clamp discreetly placed across circuit return line | Participant is expected to:  Identify high afterload and adversely impacted flows  Assess patient MAP if available  Assess return line for kinks and obstructions  Resolves obstruction and identifies return of flows to target |

**Appendix 2: Porcine Model, haemodynamic variables**
