# Supplementary material for: A training programme for novice extracorporeal resuscitation providers
Source: Resusc Plus. 2024 Jul 17;19:100720. doi: 10.1016/j.resplu.2024.100720 (PMC11301333; doi:10.1016/j.resplu.2024.100720)
Supplement: Supplementary Data 3 [file mmc3.docx]

**APPENDIX 3**

**EXTRACORPOREAL CIRCUIT MANAGEMENT: ASSESSMENT**

|  | **Yes** | **No** |
| --- | --- | --- |
| **Set up** |  |  |
| Confidently and competently sets up and primes the Cardiohelp circuit |  |  |
| Prepares the Cardiohelp console for ECMO initiation as per protocol, managing relevant interruptions to workflow from colleagues on-scene |  |  |
| Understands and what to check on the circuit, to ensure it’s safe to initiate ECMO |  |  |
| Understands what all of the buttons mean |  |  |
| Limited reliance on physical copy of protocol to achieve setup |  |  |
| Maintains asepsis and shows care to protect hardware from avoidable damage |  |  |
| **Initiation** |  |  |
| Communicates the steps of ECMO initiation clearly to the cannulation team |  |  |
| Gas is turned on at the appropriate time, as per protocol |  |  |
| Management of clamps and flows to initiate ECMO, as per protocol |  |  |
| Can determine an appropriate flow target for a given patient size |  |  |
| Quickly builds ECMO flows up to this appropriate target, or as close to the target as the circuit and physiology permits |  |  |
| Communicates flows, pressures and observed parameters in a way that allows ECMO 1 & 2 to assess the patient during upon initiation:  Maintains a consistent patter detailing the information that is required:  Coming up on flow  Flows are:  Access pressure is: |  |  |
| Can define and interpret the following parameters:   - - pVEN   - pART   - sVO2   - RPM |  |  |
| Flags with minimal delay any parameters of concern during initiation |  |  |
| **Management** |  |  |
| Shows a level of insight into ECMO patient management |  |  |
| Can integrate available information to suggest appropriate differential diagnoses of common clinical situations on ECMO |  |  |
| Displays appropriate and timely management of patients with:   - - Access insufficiency   - High afterload   - Inappropriate cannula position   - Hypertension   - Hypotension   **Noting that some of these physiological states overlap/may coexist** |  |  |
| Can identify an optimal ‘setpoint’ for a patient’s flows when limited by physiology/circuitry |  |  |
| Identifies gas failure and troubleshoots appropriately to restore oxygen delivery |  |  |
| Can perform a pump head change-out and hand-cranking procedure with confidence, minimizing no-flow time for patient and communicating with the medical team |  |  |
