# Supplementary material for: A training programme for novice extracorporeal resuscitation providers
Source: Resusc Plus. 2024 Jul 17;19:100720. doi: 10.1016/j.resplu.2024.100720 (PMC11301333; doi:10.1016/j.resplu.2024.100720)
Supplement: Supplementary Data 4 [file mmc4.docx]

APPENDIX 4: Outline of steps to proficiency for paramedic perfusionist and doctor cannulator

**Assessment/steps to proficiency**

The standards required for independent ECPR initiation have been divided into three stages:

Stage 1

- Theoretical knowledge development
- Perfusion proficiency (cardiohelp and manipulation of extracorporeal circulations)
- Proficiency in pre-hospital ECPR workflow

Stage 2: In vivo learning/experience of pre-hospital ECMO initiation with mentorship from an ECMO specialist/clinical perfusionist

Stage 3: Independent practitioner in ECMO initiation

**1) Paramedic and doctor perfusion proficiency**

The Paramedic will need to demonstrate proficiency in all of the following domains:

a) Preparation

- Establishes the “best place”, ensures circuit is off the ground and away from potential hazards, has safety equipment readily available
- Ability to anticipate and plan for potential patient deterioration when established on extracorporeal support
- Ability to be flexible and adapt the plan

b) Scene Management

- Manages both oversight of the cardiac arrest scene and initialisation of the Cardiohelp in parallel
- Communicates efficiently and respectfully with the first-on-scene crews, getting essential tasks initiated (intubation, OG tube, medication prep, etc)

c) Anticipating and planning workflow for efficiency

- Displays good time management on scene, understanding when the different phases of Cardiohelp set up need to occur based on the progress of cannulation
- Offers appropriate support to the cannulating doctors in their workflow, able to attend to requests and anticipate the next step of the cannulation process

d) Circuit skills

- Cardiohelp initiation executed with accuracy and efficiency
- 5 key safe ECMO practices **always** exhibited with no deviation from protocol (*Failure to execute any of these steps at the correct point in time will result in immediate failure of this assessment, and reassessment on another day after debrief +/-  further training*):
  - Gas delivery line connected to Cardiohelp,
  - Gas turned on at initiation
  - Heparin administered to patient
  - Tubing clamps kept near pump
  - Lines clamped in the correct place
- Independently initiates ECMO, successfully achieving the maximum flow possible for a given patient and circumstance including manipulation of clamps, RPMs and gas flow. Is not reliant upon prompts or aids
- Finds the optimal RPM setpoint for a given patient and circumstance (ie. does not ‘over-rev’)
- Maintains control throughout the initiation process – not rushing or inappropriately delaying flow delivery
- Communicates effectively with cannulating doctors throughout ECMO initiation, identifying key concerns immediately and identifying when stability is achieved
- Understands what is required to achieve stable flow and if unsure asks for help
- Ensures the Cardiohelp is not left unattended for any prolonged period, and is never left unattended in the context of clinical instability

d) Non-technical skills pertaining to:

- performance under pressure
- Ability to anticipate and plan
- Ability to mitigate anticipated risks
- Ability to openly reflect on each job as an individual and a team, to learn and improve

**2) Doctor/cannulator Proficiency**

The doctor is required to demonstrate proficiency in the following domains:

a) Preparation

- Ability to anticipate and plan for potential patient deterioration when established on extracorporeal support
- Ability to be flexible and adapt the plan

b) Demonstration of advanced vascular access skills:

- vessel access in a time pressured environment with distractors
- vessel puncture in an arrested patient +/- mCPR ongoing
- ability to insert large bore sheaths under time pressure
- demonstrate an understanding of troubleshooting common issues with cannulation

c) Ultrasound skills

- recognition of correct sono-antomy in the arrested patient
- Ability to confirm sheath wire is in vessel in the arrested patient
- Ability to visualise the IVC in the arrested patient and detect the presence of a venous wire
- Cardiac ultrasound with basic haemodynamic assessment

d) Workflow

- Anticipating and planning workflow for efficiency.
- Optimisation of the environment and equipment layout for efficient patient treatment.

Opening kit and preparing for ECMO cannulation in < 5 mins

Proficiency Assessment

Assessment of the above domains and progression in stage 2 will involve:

Step 1: Assistance with ECPR cannulation

Meeting minimum standards in the ECMO 2 role. Demonstrated ability to apply a high standard of medical knowledge to a clinical situation commensurate with the clinician’s level of training, performing the role of second cannulator, anticipating and knowing the steps of ECMO cannulation and executing appropriate wire control.

Step 2: Insertion of venous and arterial sheaths

Demonstrated ability to perform sheath/arterial line insertion commensurate with the standard expected with respect procedural success and time efficiency (< 5mins) in challenging environmental and clinical conditions.

Step 3: Venous dilation and venous cannula insertion

Demonstrated ability to successfully dilate the vessel and insert the ECMO cannula in challenging environmental and clinical conditions.
